# Mice Lacking Natural Killer T Cells Are More Susceptible to Metabolic Alterations following High Fat Diet Feeding

**DOI:** 10.1371/journal.pone.0080949

**Published:** 2014-01-20

**Authors:** Brittany V. Martin-Murphy, Qiang You, Hong Wang, Becky A. De La Houssaye, Timothy P. Reilly, Jacob E. Friedman, Cynthia Ju

**Affiliations:** 1 Department of Biotherapy, Second Affiliated Hospital, Nanjing Medical University, Nanjing, Jiangsu, China; 2 Skaggs School of Pharmacy and Pharmaceutical Sciences, University of Colorado Anschutz Medical Campus, Aurora, Colorado, United States of America; 3 Barbara Davis Center for Diabetes, University of Colorado Anschutz Medical Campus, Aurora, Colorado, United States of America; 4 Division of Endocrinology, Diabetes & Metabolism, University of Colorado Anschutz Medical Campus, Aurora, Colorado, United States of America; 5 Department of Pediatrics, University of Colorado Anschutz Medical Campus, Aurora, Colorado, United States of America; 6 Drug Safety Evaluation, Research & Development, Bristol-Myers Squibb Company, Princeton, New Jersey, United States of America; Pennington Biomedical Research Center, United States of America

## Abstract

Current estimates suggest that over one-third of the adult population has metabolic syndrome and three-fourths of the obese population has non-alcoholic fatty liver disease (NAFLD). Inflammation in metabolic tissues has emerged as a universal feature of obesity and its co-morbidities, including NAFLD. Natural Killer T (NKT) cells are a subset of innate immune cells that abundantly reside within the liver and are readily activated by lipid antigens. There is general consensus that NKT cells are pivotal regulators of inflammation; however, disagreement exists as to whether NKT cells exert pathogenic or suppressive functions in obesity. Here we demonstrate that CD1d^−/−^ mice, which lack NKT cells, were more susceptible to weight gain and fatty liver following high fat diet (HFD) feeding. Compared with their WT counterparts, CD1d^−/−^ mice displayed increased adiposity and greater induction of inflammatory genes in the liver suggestive of the precursors of NAFLD. Calorimetry studies revealed a significant increase in food intake and trends toward decreased metabolic rate and activity in CD1d^−/−^ mice compared with WT mice. Based on these findings, our results suggest that NKT cells play a regulatory role that helps to prevent diet-induced obesity and metabolic dysfunction and may play an important role in mechanisms governing cross-talk between metabolism and the immune system to regulate energy balance and liver health.

## Introduction

Inflammatory processes in metabolically-active tissues have emerged as a universal feature of obesity and its co-morbidities, including non-alcoholic fatty liver disease (NAFLD). While initial work highlighted the contribution of macrophages to tissue inflammation and insulin resistance [1]–[4], recent studies demonstrate that adaptive and other innate immune cells also participate in obesity-induced pathogenesis of these conditions [5]–[7]. However, the molecular and cellular pathways by which the immune system contributes to the control of tissue and systemic metabolism remain poorly understood.

Natural killer T (NKT) cells constitute a unique subset of immune cells that are present in various peripheral organs, such as the liver where they represent 30–50% of total liver lymphocytes [8], [9]. NKT cells express a semi-invariant T cell receptor that recognizes glycolipid antigens presented by the MHC class I-like molecule CD1d. Upon activation via the TCR, NKT cells exert their immunoregulatory functions via the production of both Th1 and Th2 cytokines, such as IL-4 and IFN-γ [10], [11]. NKT cells have been reported to protect against or contribute to a variety of diseases [12], [13]. Although a variety of NKT cell populations have been identified, including Type I, Type II, and NKT-like cells [10], [11], Type I represents the vast majority of NKT cells and is the best characterized subset in mouse and human. Type I NKT cells express a TCR composed by rearrangement of the Vα14 gene segment (or Vα24 in human) to the Jα18 segment. The loss of either CD1d or Jα18 impairs NKT cell development in the thymus and yields an NKT cell deficient mouse. Jα18^−/−^ mice are deficient only in Type I NKT cells. In contrast, CD1d^−/−^ mice lack both Type I and Type II NKT cells, as CD1d is required for positive selection of both subsets in the thymus.

Murine models of obesity (*ob/ob* mice and high-fat diet [HFD] feeding) display decreased numbers of NKT cells [14]–[16] in the liver. Further, when NKT cells are adoptively transferred into *ob/ob* mice, liver steatosis is reduced and glucose sensitivity is markedly improved [14], [15], [17], [18]. When NKT cells are stimulated by glucocerebroside, a decrease in fat accumulation in the liver has been observed [19], [20]. Conversely, an increase of NKT cells have been reported in the liver of patients with advanced NAFLD [21]. Recently it was also shown that NKT cell activation in adipose tissue of HFD-fed mice causes impairment of metabolic functions through the release of proinflammatory cytokines and the recruitment of other pathogenic immune cells [22]. Thus, both suppressive and inflammatory functions have been ascribed to NKT cells in an obese state.

The present studies add to the developing knowledge of a role for NKT cells in obesity by showing that CD1d^−/−^ mice, which lack both Type I and Type II NKT cells, are more susceptible to diet-induced obesity and metabolic perturbations. Because studies thus far used CD1d^−/−^ mice on C57Bl/6 background, we chose to study CD1d^−/−^ mice on a Balb/c background to address the impact of genetic background on the results. Notably, Balb/c mice are well known to be resistant to HFD-induced obesity [23], a finding we confirmed in WT mice. However, following high fat feeding in CD1d^−/−^ mice, we observed increased weight gain and adipose-tissue expansion compared to WT mice. Furthermore, NKT cell-deficient mice developed fatty liver, elevated liver enzymes, insulin resistance, and glucose intolerance, associated with increased appetite and an increase in inflammatory mRNA gene expression pattern. These results suggest that NKT cells play an important role in regulating susceptibility to obesity, perhaps through modulating immune cell responses during diet induced obesity.

## Materials and Methods

### Ethics Statement

All animal procedures were approved by the Institutional Animal Care and Use Committee of the University of Colorado Anschutz Medical Campus.

### Animal Care and Maintenance

Female Wild-type (WT) BALB/c and CD1d^−/−^ mice on a BALB/c background (Jackson Laboratories, Bar Harbor, ME), were maintained on a constant 12 hr light: 12 hr dark cycle with free access to water and ad libitum access to standard chow diet or HFD (58 kcal% fat with sucrose Surwit Diet, D12331, Research Diets Inc. New Brunswick, NJ). Animals were maintained under pathogen-free conditions in the Center for Laboratory Animal Care at University of Colorado Anschutz Medical Campus.

### Glucose and Insulin Tolerance Tests

All mice were fasted overnight prior to glucose tolerance test and 5 hr prior to insulin tolerance testing. Glucose and insulin tolerance tests were performed by bolus i.p. injection of glucose (1 g/kg) or insulin (0.35 U/kg), respectively. Blood glucose was measured from the tail using a glucometer (OneTouch Ultra Smart) at baseline (0) and 10, 20, 30, 45, 60, 90 and 120 min after injection.

### Metabolic Parameters

Body composition was measured on anesthetized mice by dual-energy X-ray absorptiometry using a mouse densitometer (PIXImus2; Lunar, Madison, WI). Blood was collected by retro-orbital bleed. The degree of liver injury was determined by measurement of serum ALT levels using a colorimetric assay (Teco Diagnostics, Anaheim, CA). Serum triglycerides were determined using a triglyceride GPO reagent kit (TECO diagnostics, Anaheim, CA). Extraction of tissue triglycerides was performed using chloroform∶methanol at a 2∶1 ratio, as previously described [24]. For oil red O staining [25], frozen liver sections were obtained, placed in OTC and snap-frozen in liquid nitrogen for subsequent sectioning.

### Indirect Calorimetry and Physical Activity Measurements

An open-ended indirect calorimetry system coupled with Columbus Instruments Opto M3 multichannel activity monitor was used to measured average daily food intake, oxygen consumption (O_2_) and Carbon dioxide (CO_2_) production in mice as previously described [26]. In brief, animals were placed in 4 individual metabolic chambers for measurements taken over three days with free access to food and water. Respiratory quotient (RQ) and metabolic rate were calculated and stored in a computer configured with data acquisition hardware (Analogic, Wakefield, MA) and software (Labtech, Wilmington, MA). Metabolic rate was normalized to lean body weight.

### Quantitative Real Time-PCR

Total RNA was isolated from liver using RNeasy mini kit (Qiagen), as described by the manufacturer. RNA purity and integrity were verified by measuring the ratio of absorbance at 260 and 280 nm (*A*_260_/*A*_280_). RNA (1 µg) was reverse-transcribed to cDNA using SuperScript II Reverse Transcriptase (Invitrogen, Grand Island, NY). cDNA was used for quantitative real-time PCR in conjunction with the GoTaq Gene Expression System and gene specific primers. mRNA levels were quantified by using a Real-Time PCR 7500 SDS system and software (Applied Biosystems, Carlsbad, CA).

### Liver Nonparenchymal Cell (NPC) Isolation and Flow Cytometric Analysis

Hepatic NPCs were isolated at indicated times during HFD feeding, using a previously established method [27] with slight modifications. In brief, mice were anesthetized and liver tissues were perfused *in situ* via the superior vena cava with a perfusion buffer [1X Hank's Balanced Salt Solution (HBSS) followed by *in vitro* digestion with digestion buffer [1X HBSS supplemented with 0.05% collagenase (Type IV, Sigma), 1.25 mM CaCl_2_, 4 mM MgSO_4_ and 10 mM HEPES]. Following *in vitro* digestion, the liver was disrupted in ACD solution [1X HBSS supplemented with 0.5% fetal bovine serum (FBS), 0.6% citrate-dextrose solution, 1X penicillin/streptomycin, and 10 mM HEPES] and the cells passed through a 100 µm cell strainer. The cells were centrifuged at 30× g for 3 min to pellet hepatocytes. Cells in the supernatant were then centrifuged at 320× g for 5 min, resuspended in complete RPMI media (RPMI supplemented with 10% FBS, 10 mM HEPES and 1X penicillin/streptomycin) and then fractionated using 30% (w/v) Nycodenz (Axis-Shield) at 1.155 g/mL to yield liver NPCs free of erythrocytes.

To prevent non-specific binding, liver NPCs were blocked with normal rat serum (Sigma) and anti-mouse FcãR II/III (clone 93, eBioscience). Liver NPCs were subsequently characterized by staining with the following antibodies from eBioscience: fluorescein isothiocyanate (FITC) conjugated anti-mouse CD3, phycoerythrin (PE) conjugated anti-mouse Foxp3, PE conjugated anti-mouse IL-17A, and from BioLegend: allophycocyanin (APC) conjugated anti-mouse F480, and PE-cyanine (Cy)7 conjugated anti-mouse CD11b. Seven-aminoactinomycin D (7-AAD) viability staining solution (eBioscience) was used to determine cellular viability. Cells were analyzed on a FACSCalibur cytometer (Becton Dickinson Immunocytometry System) using FlowJo 6.3.3 software (Tree Star, Inc.). For flow cytometric analysis, cells were initially gated on forward scatter (FSC) and side scatter (SSC) and then gated on live cells (7-AAD^−^).

### Immunoblot Analysis

Mice fed with HFD for 20 weeks were sacrificed, and perigonadal white adipose tissues were dissected and homogenized in lysis buffer containing protease inhibitors. Samples (30 µg) were resolved on 12% polyacrylamide gels and transferred to nitrocellulose membranes (Bio-Rad Laboratories Inc., Hercules, CA). Membranes were blocked with 5% fat-free milk and probed with antibodies against total AKT and phospho-AKT Ser 473 (1∶1,000, Cell Signaling, Danvers, MA) overnight at 4°C. Membranes were then incubated with horseradish peroxidase-conjugated goat anti-rabbit IgG antibody (1∶2,000; Jackson ImmunoResearch Laboratories, Inc. West Grove, PA) for 1 hour at room temperature. Membranes were prepared using Pierce ECL Pierce ECL Plus Western Blot Substrate (Thermo Fisher Scientific Inc. Rockford, IL) and exposed to X-ray film. The scanned bands' intensity was determined by Adobe Photoshop and the ratio of pAKT versus total AKT was calculated.

### Statistics

Statistical analysis was performed using the student's t test and one and two-way ANOVA with Bonforroni post-test. Differences in values were considered significant at *P*<0.05.

## Results

### CD1d^−/−^ Mice Are More Susceptible than WT Mice to HFD-Induced Weight Gain and Fat Accumulation in the Liver and Adipose

**Figure 1:**
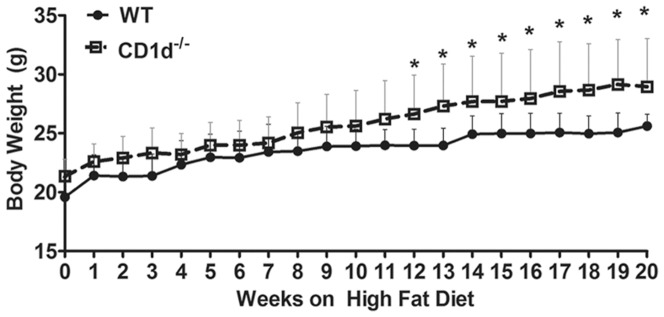
Greater daily food intake and weight gain are observed in CD1d^−/−^ mice compared with WT mice. Balb/c WT and CD1d^−/−^ mice were fed a HFD for the durations indicated and monitored for total body weight gain. * p<0.05 versus WT mice. Results from two independent experiments were combined. (n>15 mice per groups).

We found that even on regular chow diet, CD1d^−/−^ mice (21.34±1.44 g, n = 23) were significantly heavier than WT mice (19.58±1.66, n = 22) at 8 weeks of age. To compare their responses to HFD, WT and CD1d^−/−^ mice were fed a 58% Kcal HFD for 20 weeks, beginning at 8 weeks of age. Body weight of CD1d^−/−^ mice was significantly greater than WT mice, starting at 12 weeks and remaining elevated until sacrifice at 20 weeks (Figure 1).

**Figure 2:**
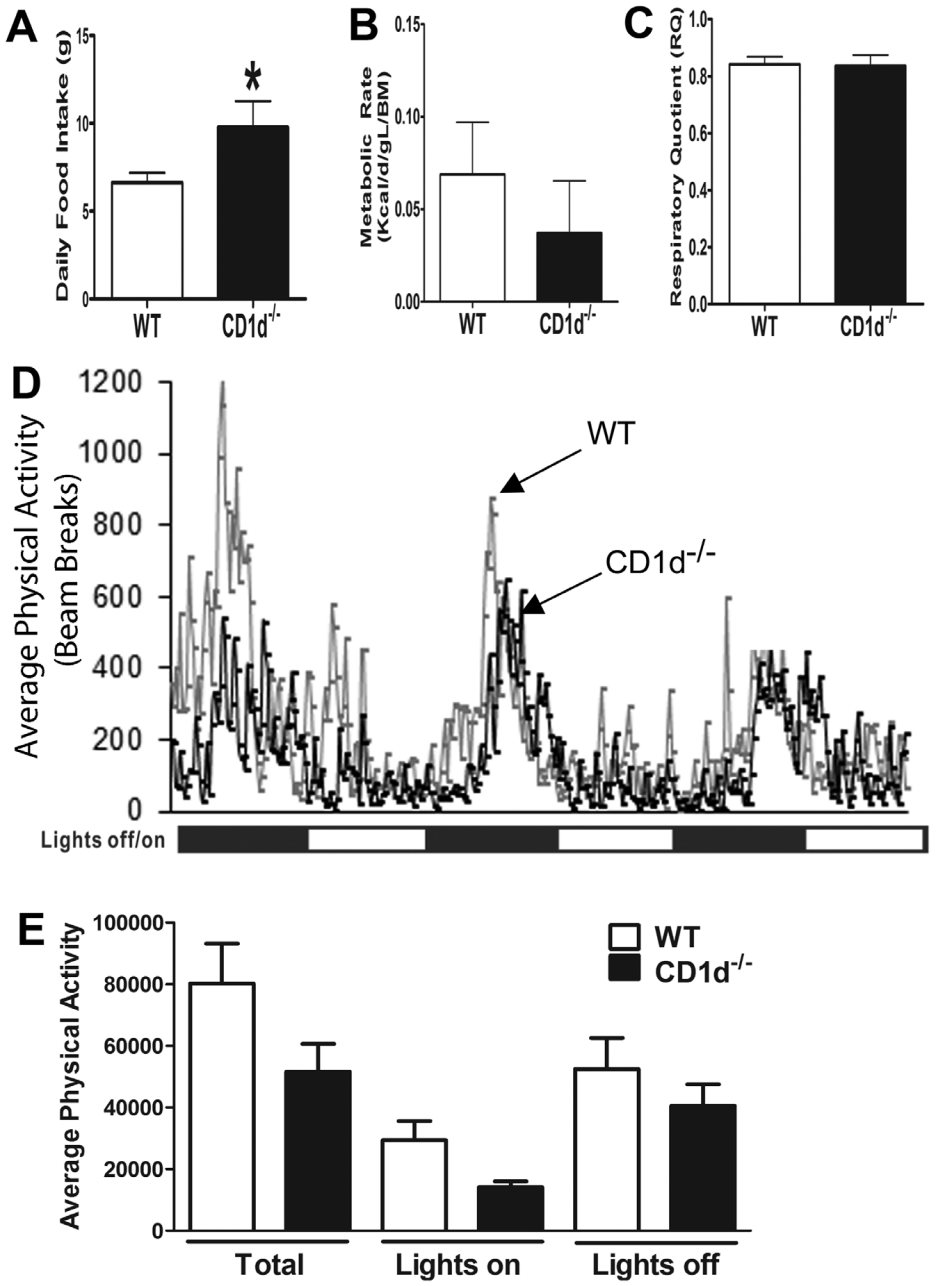
Energy balance is altered in CD1d^−/−^ mice following HFD feeding. WT and CD1d^−/−^ mice were fed HFD for 20 weeks. Average daily food intake (**A**), metabolic rate (**B**) and respiratory quotient (**C**) were measured in an indirect calorimeter. Physical activity was measured as the number of breaks in infrared beams through the 3-day calorimetry experiment (**D, E**). Results represent mean ± SEM of 6 mice per group.

To determine the reasons for increased body weight, indirect calorimetry characterization of energy balance was carried out following HFD exposure. The CD1d^−/−^ mice displayed a significantly greater average daily food intake (Figure 2A) and a trending decrease in the metabolic rate (Figure 2B) compared to WT. No significant difference was observed in the average respiratory quotient (RQ) between WT and CD1d^−/−^ mice (Figure 2C). Furthermore, CD1d^−/−^ mice displayed a trend for reduction in physical activity (Figure 2D and 2E). These data suggest that the protective role of NKT cells in obesity could be due to regulation of food intake during HFD-induced obesity.

**Figure 3:**
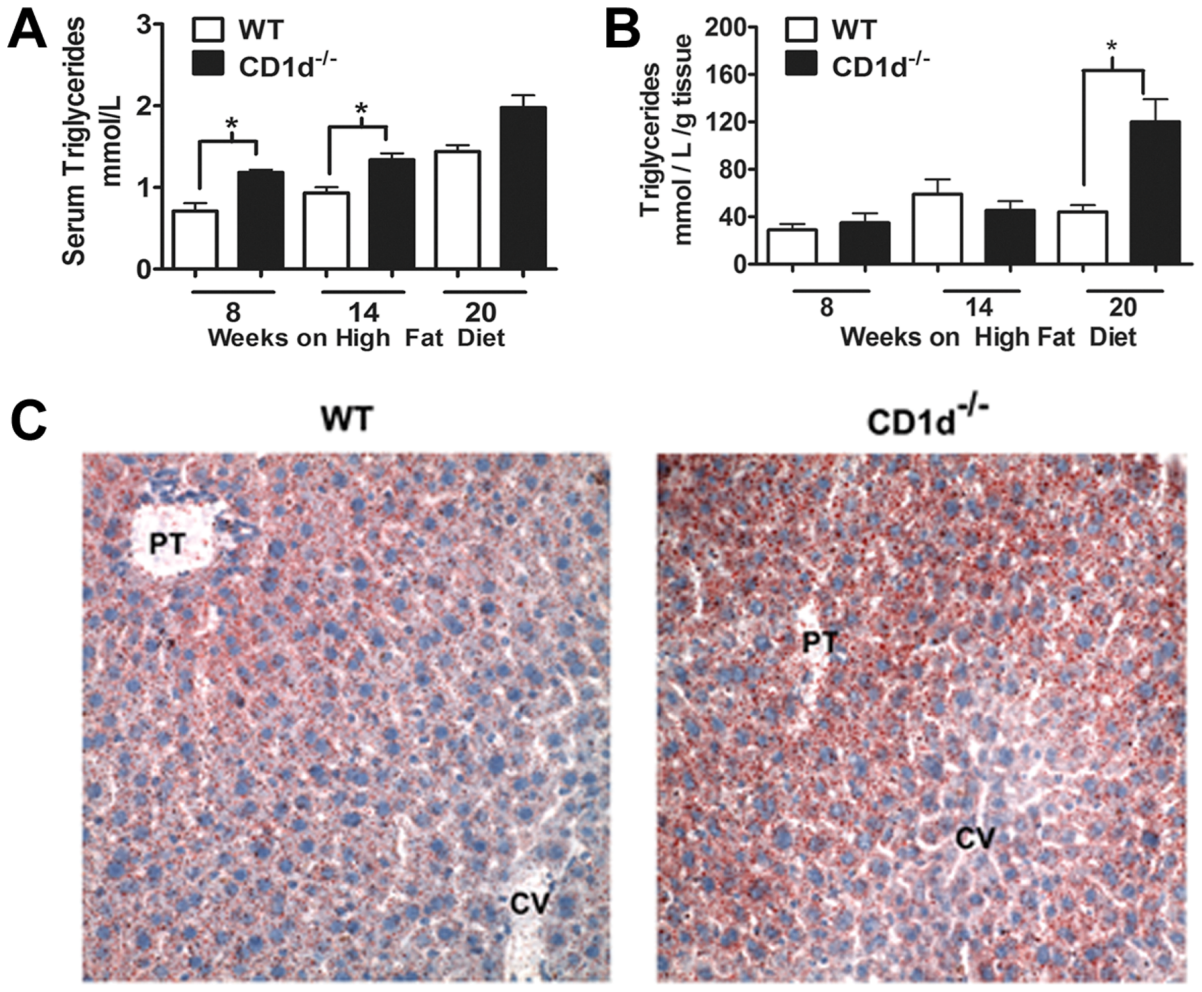
CD1d^−/−^ mice display greater increases of serum and hepatic triglycerides following HFD. WT and CD1d^−/−^ mice were fed a HFD for the durations indicated and monitored for serum (**A**) and liver (**B**) triglycerides. * p<0.05 versus WT mice. Results from two independent experiments were combined. (n>15 mice per group). Panel **C**, photomicrograph (400× final magnification) of Oil red O staining of liver sections from WT and CD1d^−/−^ mice following 20 weeks HFD feeding. CV, central vein; PT, Portal Triad.

HFD-fed CD1d^−/−^ mice showed significantly greater serum triglyceride compared to WT mice (Figure 3A) beginning at 8 weeks of age. The amount of liver triglycerides was significantly higher in CD1d^−/−^ mice than WT mice at 20 weeks (Figures 3B and 3C). This result is consistent with previous report of increased hepatic accumulation of triglyceride in NKT-deficient mice [16].

**Figure 4:**
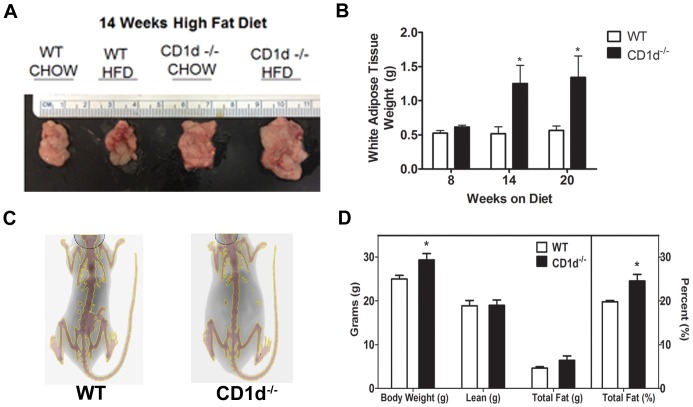
CD1d^−/−^ mice display greater WAT and total percent fat compared to WT mice. WT and CD1d^−/−^ mice were fed HFD for the durations indicated whereupon mice were sacrificed and WAT removed to measure size (**A**) and total tissue weight (**B**). Body composition and fat mass was measured by DEXA following 20 weeks on HFD (**C, D**). * p<0.05 versus WT mice. Results represent mean ± SEM of 6 mice per group.

Balb/c mice are well known to be resistant to HFD-induced obesity [23]. Consistent with this, we observed a lack of significant increase in WAT volume in WT mice on HFD (Figure 4A). Remarkably, CD1d^−/−^ mice on a HFD showed a significant increase in adipose tissue mass (Figure 4B, 4C). Dual x-ray lean absorptiometry (DEXA) revealed that CD1d^−/−^ mice had a significantly greater total and percent fat compared to WT mice, while the lean body mass was similar (Figure 4D).

### CD1d^−/−^ Mice Are More Susceptible than WT Mice to HFD-Induced Metabolic Alterations

**Figure 5:**
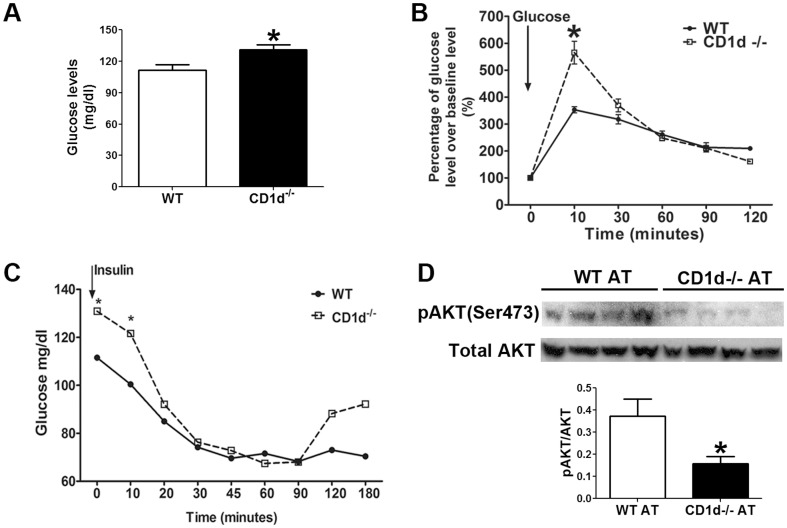
CD1d^−/−^ mice show increased glucose intolerance and insulin resistance compared to WT mice. WT and CD1d^−/−^ mice were fed a HFD for 20 weeks. Fasting glucose levels (A), GTT (**B**) and ITT (**C**) were compared. Results from two independent experiments were combined. (n≥7 mice per group). (**D**) pAKT and total AKT expression levels were determined in white adipose tissues of 4 mice per group by Western blot. The ratio of pAKT/AKT was calculated and compared between WT and CD1d^−/−^ mice. * p<0.05 versus WT mice.

After 20 weeks of HFD feeding, CD1d^−/−^ and WT mice were tested for insulin resistance and glucose tolerance. Fasting glucose levels were increased by 22% in CD1d^−/−^ mice compared with WT mice (Fig. 5A). Insulin tolerance testing (ITT) revealed a significantly greater fasting glucose levels in CD1d^−/−^ mice compared to WT mice (Figure 5C), a common phenotype seen in diabetic animals and patients with insulin resistance. It is noteworthy that serum glucose levels were higher in CD1d^−/−^ mice than WT mice during the first 10 min after an i.p. injection of insulin and also showed a rebound effect at 120 min post-insulin injection. Upon GTT, the CD1d^−/−^ mice displayed a slower rate of glucose clearance, suggesting the development of glucose intolerance (Figure 5B). We then examined insulin signaling and compared the extents of AKT activation in the adipose tissues of WT and CD1d^−/−^ mice. The levels of pAKT and the ratio of pAKT/total AKT are significantly reduced in CD1d^−/−^ mice (Figure 5D). The data suggest that insulin sensitivity is impaired in CD1d^−/−^ mice. Together, these data suggest the possible involvement of both peripheral and hepatic insulin resistance.

### Cellular Analysis of Liver and White Adipose Tissue (WAT) in WT and CD1d^−/−^ Mice

**Figure 6:**
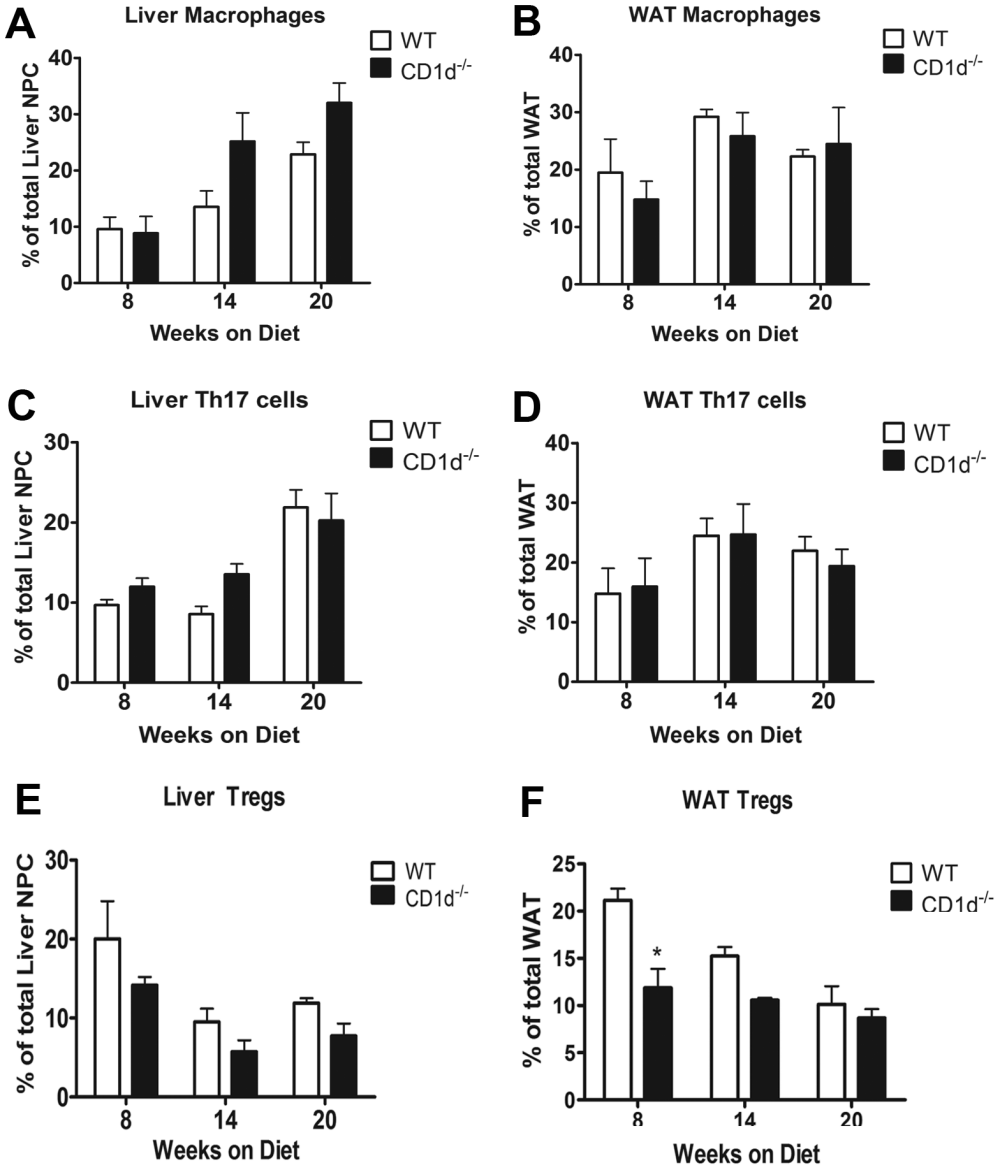
Cellular analysis of the liver and WAT in WT and CD1d^−/−^ mice following HFD feeding. Cells were isolated from the liver and WAT of WT and CD1d^−/−^ mice following 20 weeks HFD feeding. The cells were stained with anti-CD11b PE and anti-F480 antibodies to identify macrophages, anti-CD3 and anti-IL-17 to identify Th17 cells, and anti-CD3 and anti-Foxp3 to identify Treg. Quantification of liver and WAT macrophages (**A and B**), Th17 cells (**C and D**) and Treg (**E and F**) are shown. * p<0.05 versus WT mice. Results represent mean ± SEM of 3 mice per group.

Insulin resistance is believed to be initiated by a low grade systemic inflammatory process that occurs in obesity. It is well documented that the number of macrophages within the adipose tissue increases in obesity and that these cells participate in the inflammatory process and pathogenesis of metabolic disorders [1], [3], [7]. We observed trends of increase in the percentage of macrophages in the liver at 14 and 20 weeks of HFD feeding of CD1d^−/−^ mice compared to WT mice (Figure 6A). However, the number of macrophages did not appear to change in the WAT of either WT or CD1d^−/−^ mice (Figure 6B).

The pro-inflammatory nature of Th17 cells has been implicated in the pathogenesis of metabolic disorders [28]–[30]. While we did not observe any significant difference in Th17 cells in the liver between WT and CD1d^−/−^ mice at 8, 14 or 20 wks following HFD (Figure 6C), HFD feeding appeared to cause an increase in the percentage of Th17 cells in both WT and CD1d^−/−^ mice compared to the Th17 percentage in standard chow-fed mice (5%, data not shown).

HFD feeding caused a decrease of the percentage of regulatory T cells (Treg) in the liver of both WT and CD1d^−/−^ mice (Treg was 20% in naïve liver). However, there was no significant difference in liver Treg between WT and CD1d^−/−^ mice (Figure 6E). In contrast, the percentage of Treg in WAT was significantly lower in CD1d^−/−^ mice than WT mice at 8 weeks following HFD (Figure 6F). This decrease in Treg is consistent with published reports of decreased number of Treg in obese animals and patients [6].

### Comparison of Hepatic and WAT Gene Expression in WT and CD1d^−/−^ Mice

**Figure 7:**
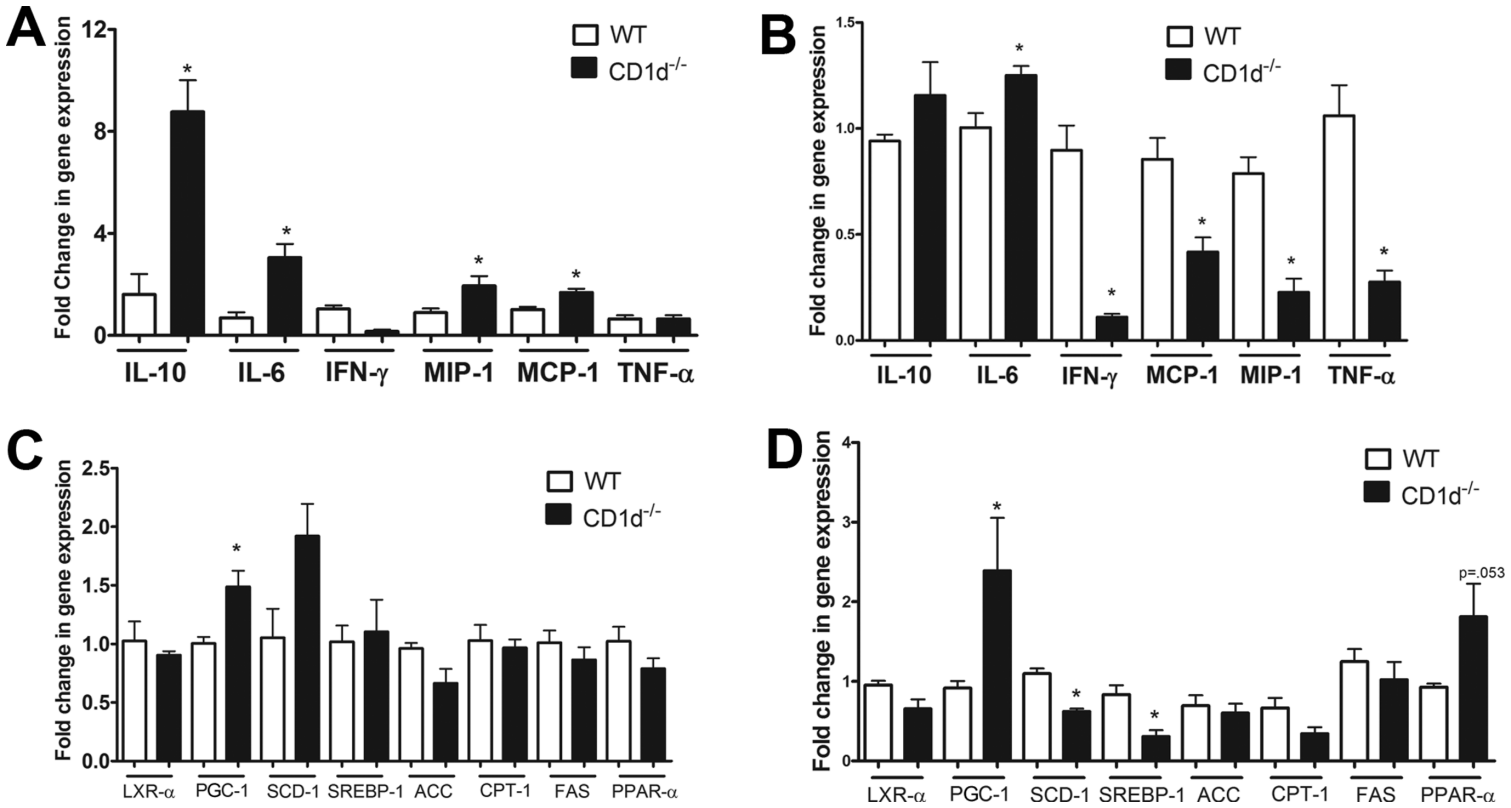
Comparing mRNA expression levels of inflammation- and metabolism-related genes in the liver and WAT of WT and CD1d^−/−^ mice. mRNA levels of various cytokines and chemokines (**A and B**) and metabolic genes (**C and D**) in the liver (**A and C**) and WAT (**B and D**) were measured by qPCR. Fold changes compared with WT mice are shown. * p<0.05 versus WT mice. Results represent mean ± SEM of 4 mice per group.

To further explore whether an altered inflammatory profile was present in the liver and WAT of CD1d^−/−^ mice following 20 weeks HFD feeding, we examined the gene expression of a variety of cytokines and chemokines. Consistent with our metabolic data showing insulin resistance, the levels of IL-6 in the liver and adipose tissue were higher in CD1d^−/−^ mice than WT mice (Figures 7A, B). We further observed a significant increase in monocyte chemotactic protein (MCP)-1 and macrophage inflammatory protein (MIP)-1 in the liver of CD1d^−/−^ mice (Figure 7A). This data correlates with the trending increase in macrophage infiltration observed in the liver of CD1d^−/−^ mice following 14 and 20 weeks HFD. Markedly greater levels of the anti-inflammatory cytokine IL-10 were also observed in the liver of CD1d^−/−^ mice, which may serve as an adaptive mechanism to suppress inflammation (Figure 7A) [31]. Interestingly, in contrast to the liver, with the exception of IL-6, all inflammatory mediators, such as IFN-γ, MCP-1 and MIP-1 were decreased in the WAT of CD1d^−/−^ mice compared to WT (Figure 7B). This decrease in inflammatory cytokines and chemokines in the WAT of CD1d^−/−^ mice may be an adaptive response. Liver steatosis is characterized by increased hepatic insulin resistance and a shift to increased lipogenesis. As such, we sought to examine the genes involving de-novo liver lipogenesis. We observed a significant increase in peroxisome proliferator-activated receptor gamma coactivator (PGC-1) in the liver of CD1d^−/−^ mice compared to WT (Figure 7C). PGC-1 is a transcriptional co-activator important in the induction of peroxisome proliferator-activated receptor (PPAR)-α expression and increased mitochondrial β-oxidation, but may also play an important role in gluconeogenesis. We also observed a trend of increase in stearoyl-coenzyme A desaturase 1 (SCD-1) in the liver of CD1d^−/−^ mice compared to WT. SCD-1 is the rate limiting enzyme involved in the conversion of saturated fatty acids to monounsaturated fatty acids (Figure 7C). The increases of PGC-1 and SCD-1 are consistent with an insulin resistant phenotype in the liver [32] and correlate with the more severe insulin resistance observed in CD1d^−/−^ mice following HFD feeding. In contrast to the insulin resistance found in the liver of CD1d^−/−^ mice, the WAT displayed an insulin sensitive phenotype with marked increase in PGC-1 and slight increase in PPAR-α (Figure 7D). Furthermore, a significant decrease in SCD-1 and sterol regulatory binding protein-1 (SREBP-1), a protein indirectly required for cholesterol and fatty acid biosynthesis, was observed in the WAT of CD1d^−/−^ mice (Figure 7D).

## Discussion

Our data indicate a regulatory role of NKT cells in preventing diet-induced obesity and its metabolic consequences. Even on an obesity resistant Balb/c background, CD1d^−/−^ mice lacking both Type 1 and Type 2 NKT cells were susceptible to HFD-induced increases in food intake and body weight, WAT accumulation, glucose intolerance and insulin resistance. The animals also demonstrated decreasing trends in metabolic rate and activity. Such observations are suggestive of a role for NKT cells in modulation of appetite regulation and energy balance. The exact mechanisms whereby NKT cells regulate immune responses and energy homeostasis are unknown; however, mounting evidence suggests that HFD affects immune cells in multiple tissues, including the hypothalamus [33] and the microbiota in the gut, with consequences on energy balance [34]. Together with evidence that NKT cell-deficient mice show increased liver inflammatory genes and steatosis, we suggest that NKT cells play a potentially broad role in modulating HFD-induced metabolic disorders, particularly in the liver but perhaps in other tissues as well.

The role of NKT cells in metabolic disease has been examined through the utilization of either CD1d^−/−^, which lack both Type I and Type II NKT cells, or Jα18^−/−^ mice which lack only the Type I NKT cells. However, results from these studies are controversial. Some studies revealed no difference or only marginal differences in lipid accumulation, glucose tolerance and insulin resistance between NKT cell-deficient and WT mice following HFD feeding [16], [35]. In contrast, other studies demonstrated increased liver triglyceride content, increased liver injury and inflammation following HFD feeding [18], [36]. These discrepancies may be due to the influence of mouse genetic background and susceptibility to metabolic disease, types of diets, and/or the fact that Type I and Type II NKT cells exhibit differing roles as well. A summary of the previous studies examining the role of NKT cells in diet-induced obesity can be found in Table 1 and 2. A few reports that have used both Ja18^−/−^ and CD1d^−/−^ mice on a C57Bl/6 background demonstrated no difference or only marginal differences in lipid accumulation, and mild to moderate changes in glucose tolerance and insulin resistance between NKT cell-deficient and WT mice following HFD [16], [35]. Whereas these results suggested that NKT cells have a limited role or no effect in HFD-induced metabolic disorders [16], [35], two other recent studies using the same mouse model demonstrated NKT cell-deficient mice to have reduced body weight, smaller adipocytes, milder hepatosteatosis, and improved glucose sensitivity and insulin tolerance compared to WT mice following HFD feeding [22], [37]. These studies concluded that NKT cells play a pathogenic role in the development of diet-induced obesity, inflammation, and insulin resistance. In contrast, the most recent publications by [18] demonstrated that compared with WT mice, NKT cell deficient mice gained more body weight, accumulated more lipids in the liver and WAT, and exhibited worsened glucose intolerant and insulin resistant [18]. Consistent with the protective role of NKT cells in metabolic disorders, it has been reported that activation of these cells with α-galactosylceramide causes macrophage polarization to an M2 anti-inflammatory phenotype and improves glucose sensitivity [18], [38]. Consistent with the protective function of NKT cells, another study by Miyagi et al. also showed increased liver triglyceride content, increased liver injury and inflammation in Jα18^−/−^ mice compared to WT mice following high fat feeding [36]. In contrast to the majority of the published studies using CD1d^−/−^ and Jα18^−/−^ on a C57Bl/6 background, the study by Miyagi et al used Jα18^−/−^ mice on Balb/c background.

**Table 1:** Role of NKT cells in diet-induced obesity, summary of previously published work.

Paper	Mouse model	Mouse back-ground	Gender	Diet % Kcal Fat	Change in					
					Body Weight	Total % Fat	Activity	Food Intake	Liver lipids	(WAT) obesity
(Satoh et al., 2012) [37]	CD1d^−/−^	C57	male	32%	CD1d^−/−^ <WT	CD1d^−/−^ <WT	N/A	No	CD1d^−/−^ <WT	CD1d^−/−^ <WT
	Jα18^−/−^	C57	male	32%	No difference	No	N/A	No	No	No
(Wu et al., 2012) [22]	CD1d^−/−^	C57	male & female	60%	CD1d^−/−^ <WT	Jα18^−/−^ <WT	No	No	CD1d^−/−^ <WT	N/A
	Jα18^−/−^	C57	male & female	60%	Jα18^−/−^ <WT	Jα18^−/−^ <WT	No	No	Jα18^−/−^ <WT	Jα18^−/−^ <WT
(Miyagi et al., 2010) [36]	Jα18^−/−^	Balb/c	male	57%	No difference	N/A	N/A	N/A	Jα18^−/−^ >WT	N/A
(Kotas et al., 2011) [16]	CD1d^−/−^	C57	N/A	60%	No difference	No	No	No	CD1d^−/−^ >WT	N/A
	Jα18^−/−^	C57	N/A	60%	No difference	N/A	N/A	N/A	No	N/A
(Mantell et al., 2011) [35]	CD1d^−/−^	C57	N/A	44%	No difference	No	No	N/A	No	N/A
(Lynch et al., 2012) [18]	CD1d^−/−^	C57	male	60%	CD1d^−/−^ >WT	CD1d^−/−^ >WT	N/A	No	CD1d^−/−^ >WT	CD1d^−/−^ >WT
	Jα18^−/−^	C57	male & female	60%	Jα18^−/−^ >WT	Jα18^−/−^ >WT	N/A	No	Jα18^−/−^ >WT	Jα18^−/−^ >WT

**Table 2:** Role of NKT cells in diet-induced obesity, summary of previously published work.

Paper	Mouse	Insulin Resistance	Glucose Intolerance	Cell infiltration		Cytokine and Chemokine		NKT role in obesity
				Liver	WAT	Liver	WAT	
(Satoh et al., 2012) [37]	CD1d^−/−^	CD1d^−/−^ <WT	CD1d^−/−^ <WT	NKT in WT	NKT in WT	TNF-a and IL6 in WT	N/A	**Pathogenic**
	Ja18^−/−^	No	No	No	No	No	N/A	**No role**
(Wu et al., 2012) [22]	CD1d^−/−^	CD1d^−/−^ <WT	N/A	N/A	N/A	N/A	N/A	**Pathogenic**
	Ja18^−/−^	Ja18^−/−^ <WT	N/A	N/A	Macro in WT	TNF-a, MCP-1, In WT	TNF-a, MCP-1, in WT	
(Miyagi et al., 2010) [36]	Ja18^−/−^	N/A	N/A	CD4^+^ T CD8^+^ T In KO	N/A	TNF-a, IFN-g, IL-10, MIP-2, IP-10, In KO	N/A	**Protective**
(Kotas et al., 2011) [16]	CD1d^−/−^	No	CD1d^−/−^ >WT	N/A	N/A	N/A	N/A	**Minimal protective**
	Ja18^−/−^	No	No	N/A	N/A	N/A	N/A	**No Role**
(Mantell et al., 2011) [35]	CD1d^−/−^	No	No	No	No	No	No	**No Role**
(Lynch et al., 2012) [18]	CD1d^−/−^	No	CD1d^−/−^ >WT	N/A	Macro in CD1d^−/−^	N/A	Increased CD11c, decreased CD206 in CD1d^−/−^	**Protective**
	Ja18^−/−^	Ja18^−/−^ >WT	Ja18^−/−^ >WT	N/A	Macro in Ja18^−/−^	N/A	Increased CD11c, decreased CD206 in Ja18^−/−^	**Protective**

It has been reported recently that macrophage numbers and M1 polarization in CD1d^−/−^ mice are increased if compared with their WT counterparts under HFD conditions [39]. However, these changes were not observed in our study. This discrepancy may be due to two main differences between the two studies. First, the CD1d^−/−^ mice used in our study are on Balb/cJ background but the CD1d^−/−^ mice used in the JCI study are on C57B6 background. It has been shown that C57B6 mice are prone to develop obesity and insulin resistance than Balb/c mice [40]. Thus, it is possible that compared with CD1d^−/−^ -Balb/c mice, the CD1d^−/−^ -C57B6mice develop a more severe phenotype with not only worsened insulin resistance but also increased M1-mediated inflammation. Second, the HFD used in the two studies is different. We used Research Diet D12331, and the JCI study used D12451. It has been shown that another type of HFD, D12492, induces greater insulin resistance than D12331 in C57BL/6 mouse due to lard, sodium level and fiber differences [41]. Although D12451 and D12331 have not been compared side-by-side, lard is present in D12451, but not in D12331.

Balb/c and C57Bl/6 mice are two widely used strains throughout biomedical research. These two strains of mice have demonstrated different responses to drug-induced liver injury [42], induction of autoimmune diseases [43], and responses to HFD diet. For example, Balb/c mice have been shown to be resistant to adenovirus type 1 [44] and measles virus [45] whereas C57Bl/6 are more susceptible. Conversely, Balb/c mice are more susceptible to herpes simplex virus [46] and *Leishemonia* [47] and C57Bl/6 are resistant. The difference in susceptibilities to pathogens is hypothesized to be due to the different Th1/Th2 responses in these mice following infection. T cells from C57Bl/6 mice have been shown to preferentially produce Th1 cytokines, such as IFN-γ; whereas, T cells from Balb/c mice produce Th2 cytokines with the favored production of IL-4 [48], [49]. Further, isolated splenic DX5^+^ NKT cells from Balb/c and C57Bl/6 mice displayed differential cytokine production following stimulation, with Balb/c producing more Th2 cytokines IL-10 and IL-13 than C57Bl/6J mice [50].

Previous research demonstrates that Balb/c mice express a population of thymic NKT cells that are greater in number and express higher levels of IL-4 compared to C57Bl/6 mice [51]. This may be particularly important in models of obesity, as IL-4 has been linked to protection from metabolic dysregulation. Previous studies have shown IL-4 activation of STAT6 in hepatocytes as important in regulating fatty acid oxidation through the suppression of PPAR-α [52]. Recent work has also shown that IL-4 increases thermogenic gene expression, fatty acid mobilization and energy expenditure, by means of stimulating alternatively activated macrophages [53]. Further, a recent study demonstrated that IL-4 produced by eosinophils, in the visceral adipose tissues is important in protecting mice from HFD-induced obesity, through the maintenance of alternatively activated macrophages [5]. Another report revealed that 4 days of HFD feeding of WT mice was enough to activate NKT cells to produce IL-4, which activated M2 macrophages; however, IL-4 expression was much lower in CD1d^−/−^ mice [54]. These studies suggest that IL-4 produced by NKT cells is an important inhibitor of inflammation, glucose intolerance and insulin resistance. Given the protective role of IL-4 in metabolic regulation we speculate, that in the absence of NKT cells this protection is lost, thus lending NKT cell-deficient mice more susceptible to HFD-induced obesity and metabolic dysfunction. We hypothesize that because the difference of IL-4 levels between WT and NKT cell deficient mice on Balb/c background is greater than the difference between WT and NKT cell deficient mice on C57Bl/6 background, we observed a more severe phenotype in CD1d^−/−^-Balb/c mice following HFD feeding, compared with other studies of CD1d^−/−^-B6 mice. Among published reports thus far, 2 studies [16] comparing WT mice with CD1d^−/−^-B6 showed similar but milder phenotype in CD1d^−/−^-B6 mice than what we observed. Kotas et al showed that CD1d^−/−^-B6 mice to be more glucose intolerant but mildly insulin resistant compared to WT following HFD feeding; however, body weight gain, liver and WAT lipid accumulation were not different between CD1d^−/−^-B6 and WT mice [16]. Lynch et al. showed higher weight gain, higher liver and WAT lipid accumulation, and more severe glucose intolerance in CD1d^−/−^ -B6 than WT mice; however, there was no difference in insulin resistance between WT and CD1d^−/−^-B6 mice [18].

Elevated inflammation has been hypothesized as an important link in the development of insulin resistance. Activated NKT cells are capable of activating other innate and adaptive immune cells and driving both anti-inflammatory and proinflammatory responses, as well as regulating the hepatic recruitment of other types of immunoregulatory cells. We observed trending increase in macrophage accumulation in the liver of CD1d^−/−^ mice over time compared to WT (Figure 6A), and a greater loss of Treg cells in liver and adipose tissue (Figure 6E and 6F). These data suggest that the lack of NKT cells may contribute to activity of other immune cells that contribute to subsequent development of inflammation and fatty liver. We found an elevation of IL-6 mRNA in the liver and adipose tissue of CD1d^−/−^ compared to WT mice (Figures 7A, B). This data is consistent with a diabetic phenotype, as elevated levels of IL-6 in the sera, liver, and WAT correlate with greater BMI, and insulin resistance [55], [56]. Further, IL-6 has been shown to enhance circulating triglycerides and glucose levels [57], [58]. Contrary to these reports, IL-6^−/−^ mice develop a mature onset obesity that can in part be reversed by replacing IL-6 [59], suggesting an important therapeutic window for IL-6 levels that may be beneficial compared to levels linked with disease. We further observed a significant increase in MCP-1 and MIP-1 in the liver of CD1d^−/−^ mice (Figure 7A). This data support a previous report by Miyagi et al. which also described elevated MIP-1 levels in the liver of CD1d^−/−^ mice following HFD-feeding [36]. Surprisingly, qPCR analysis of the WAT revealed a significant decrease in IFN-γ, MCP-1 and MIP-1 in CD1d^−/−^ mice compared to WT (Figure 7B), which may reveal an adaptive response of CD1d^−/−^ mice. Overall, the majority of proinflammatory genes were not dramatically higher in the adipose tissues of CD1d^−/−^ mice. This may suggest that inflammation is not the major cause, but rather a result or parallel change, of HFD-induced metabolic disorder in CD1d^−/−^ mice in our model.

In addition, it has been reported that NKT cell deficiency in Nonobese Diabetic (NOD) mice contributes to the susceptibility of NOD mice to autoimmune type 1 diabetes [60] [61]. The absence of NKT cells confers worsened type 1 diabetes, whereas either an increase of NKT cell number or NKT cell activation mitigates the disease [62]. The possible impact of NKT cell deficiency on beta cell function in the model of HFD-induced insulin resistance warrants further investigation in future studies.

In summary, our studies demonstrated that depletion of both Type I and Type II NKT cells resulted in an obese phenotype with insulin resistance, glucose intolerance, body weight gain, and lipid accumulation in the liver and WAT following HFD feeding. Although there have been several studies investigating the role of NKT cells in diet-induced obesity, the findings have been controversial and conflicting. We observed a more dramatic phenotype in NKT cell-deficient mice on Balb/c background, and our findings support a protective role of these cells against development of insulin resistance and metabolic disorders. Although the majority of the published studies focus on the immune modulatory function of NKT cells as an explanation of their role either protect or exacerbate diet-induced obesity, our data revealed that CD1d^−/−^ mice are significantly more susceptible to HFD feeding due to excessive energy intake and a trending reduction in physical activity. Further studies are warranted to elucidate the molecular mechanisms involving the role of NKT in the regulation of appetite suppression and overall protection from diet induced obesity.
